# Design and methodology of a cluster randomized factorial trial to optimize implementation strategies for the Healthy School Recognized Campus initiative

**DOI:** 10.3389/fpubh.2025.1652485

**Published:** 2025-10-30

**Authors:** Jacob Szeszulski, Allyson Schaefers, Gabrielli T. De Mello, Julie Gardner, Alisha George, Alexandra MacMillan Uribe, Chad D. Rethorst, Rebecca A. Seguin-Fowler, Lucy Xin

**Affiliations:** ^1^Department of Nutrition, Texas A&M AgriLife Research, Institute for Advancing Health Through Agriculture (IHA), Dallas, TX, United States; ^2^Family and Community Health, Texas A&M AgriLife Extension, College Station, TX, United States; ^3^Texas College of Osteopathic Medicine, University of North Texas, Fort Worth, TX, United States

**Keywords:** exercise, healthy diet, program evaluation, metabolic syndrome, metabolic diseases, implementation science, schools

## Abstract

**Background:**

The Healthy School Recognized Campus initiative bundles multiple school- and research-based programs for children and adults to improve physical activity and nutrition outcomes that affect cardiovascular disease risk. This study aims to test the individual and combined impact of three implementation strategies on implementation and effectiveness outcomes.

**Methods:**

Using the Multiphase Optimization STrategy (MOST) framework and a cluster randomized full factorial study design, two cohorts (*n* = 8; *n* = 16 total) of public elementary schools in North and East Texas will be randomized to receive combinations of the three implementation strategies – additional resources, school-to-school mentoring, and enhanced engagement – over one academic year. We will survey program implementers (e.g., Extension agents, school staff, administrators) to determine the dose of the Healthy School Recognized Campus initiative that each student receives. We will objectively measure changes in students’ MetS risk, cardiovascular fitness measured via the Progressive Aerobic Cardiovascular Endurance Run, dermal carotenoids (an estimate of fruit and vegetable intake) measured via the Veggie Meter, and body mass index pre- and post-intervention. The individual and combined (e.g., synergistic, antagonistic) impact of strategies will be evaluated after each cohort using a general linear model framework, and strategies will be modified and prepared for testing in a future randomized controlled trial.

**Discussion:**

By using rigorous implementation science frameworks, developing three implementation strategies, and evaluating implementation and effectiveness outcomes, this study aims to determine which implementation strategy or combination of strategies have the biggest impact on the Healthy School Recognized Campus initiative.

**Trial registration:**

Registered at clinicaltrials.gov on August 2nd, 2023 (NCT05977959).

## 1 Introduction

In the United States, approximately one in three youth are overweight, and 85% of overweight youth have at least one metabolic syndrome (MetS) risk factor that increases their chance of developing chronic diseases (e.g., cardiovascular disease, diabetes) (1, 2). In 2000, the prevalence of MetS in the United States was about 6.4%; however, the prevalence rate among obese children has been found to be as high as 50% (3). Given that the rate of body mass index (BMI) acceleration doubled during the COVID-19 pandemic, the prevalence of MetS in youth is also likely to increase in the coming years (4).

Established by the U. S. Department of Health and Human Services, The Community Preventive Services Task Force has identified the delivery of school based physical activity and nutrition evidence-based programs (EBPs) as an effective approach for improving obesity, and consequently, MetS and other chronic disease risk factors among youth (5–8). Schools are also a critical setting to improve health behaviors (i.e., physical activity and nutrition), as almost 57 million U. S. children and adolescents attend school, spending an average of 6.5 h there each day (9). Experts recommend that schools help students achieve 30 min of daily physical activity, eat ≥2 vegetables, and a fresh fruit each day, but these goals are rarely met (10, 11). Accordingly, the delivery of more EBPs that promote physical activity and fruit and vegetable consumption are essential for changing youths’ MetS risk.

One way to improve the number of EBPs delivered is the use of bundling approaches or care bundles, whereby a set of evidence-based practices or programs are implemented at the same time in coordination with one another (12, 13). Theoretically, the use of bundled approaches should improve behavioral or health outcomes better than a single program alone; however, most bundled approaches have been tested in health care settings, and results from these studies show mixed or inconclusive findings (13). In the school setting, previous research has demonstrated that for each additional physical activity or nutrition program that a school implemented, the odds were 4% higher for students meeting recommended standards for BMI (14). The use of bundled approaches is also highly relevant for school settings, as most schools are already delivering multiple physical activity (e.g., recess, physical education, after school programs) and/or nutrition programs (e.g., health education, cooking classes) concurrently, and bundled approaches align with conceptual frameworks for improving multiple aspects of student health (e.g., Whole School, Whole Community, Whole Child framework) (15–18).

Still, schools face critical challenges for concurrently delivering multiple EBPs. For example, one systematic review identified 22 different barriers that affect schools’ willingness to implement EBPs (e.g., time, availability/quality of resources, school climate) (19). Further, barriers/facilitators to program implementation are often specific to a staff member’s role within the project (e.g., teachers report resources as a barrier, whereas principals report staff turnover) (20, 21), the school’s context, or the EBPs being delivered, which necessitates the development of optimized implementation strategies (i.e., methods tailored to specific implementation barriers) (22, 23). Optimization is the process of balancing intervention effectiveness with its ability to be implemented (e.g., affordability, scalability, efficiency) (24, 25). Accordingly, implementation strategies that are optimized to support the delivery of bundled EBPs in schools can improve the number and quality of EBPs implemented, and ultimately, health outcomes (26). For example, one study found that when school staff perceived successful implementation of physical activity and nutrition programs, students were 32% more likely to meet recommended standards for cardiovascular fitness (14).

Accordingly, we aim to improve the implementation and effectiveness of the Healthy School Recognized Campus (HSRC) initiative, which promotes the delivery of physical activity and healthy eating programs by bundling AgriLife Extension’s school- and research-based programs. For a school to be recognized as a HSRC, they are required to host a school-wide walking challenge to increase students’ physical activity (i.e., Walk Across Texas), at least one other AgriLife Extension program for students, and at least one AgriLife Extension program for adults. The aims of this study are to describe the protocol for a cluster randomized factorial trial to: (1) evaluate the individual and combined (synergistic or antagonistic) impact of three implementation strategies on the delivery of bundled physical activity and nutrition programs and (2) evaluate the effectiveness of a bundled approach on improving MetS among elementary school-aged children.

## 2 Materials and methods

### 2.1 Study setting

We will recruit two cohorts of eight schools from the North and East regions of Texas to participate in this study. Texas A&M AgriLife Extension defines North and East Texas as a 44-county service area. From west to east, this region includes Cooke, Denton, and Tarrant counties – which includes the Dallas-Fort Worth metroplex – and all other Texas counties east of the Trinity River. From north to south this region includes counties along the Oklahoma border, and extends south to Jasper/Newton counties (a full list of counties can be found at agrilifeextension.tamu.edu/about-2/district-offices-regional-centers/).

Counties in the North and East Texas AgriLife regions have some of the highest rates of cardiovascular disease in the country; and the prevalence of MetS is around 9%. The average rate of cardiovascular disease in these 44 counties is 556.3 deaths per 100,000 people over 18 years of age, which is substantially higher than the average of all Texas counties (442.5 per 100,000 people) and the average of all counties in the United States (432.3 per 100,000 people) (27). The rates in these counties are also higher for deaths from heart disease (430.6 vs. 334.5 in Texas vs. 325.7 in the United States), deaths from stroke (92.14 vs. 80.1 in Texas vs. 75.7 in the United States), and preventable/avoidable deaths from cardiovascular diseases (403.4 vs. 324.6 in Texas vs. 310.3 in the United States) (27). Similarly, these counties also have a higher average prevalence of cardiovascular disease risk factors than the average of other Texas counties, including for high blood pressure (39.1% vs. 37.4% in Texas), high cholesterol (40.0% vs. 39.6% in Texas), diabetes (8.2% vs. 7.9% in Texas), obesity (23.1% vs. 21.4%) leisure time physical inactivity (17.7% vs. 16.1%) and smoking (18.3% vs. 17.0%) (27).

### 2.2 Study design

We will examine how three discrete implementation strategies affect important implementation and cardiovascular disease outcomes using the Multiphase Optimization STrategy (MOST) framework and a cluster randomized factorial design. The cluster randomized full factorial design allows us to calculate effect estimates of each individual strategy, as well as all combinations of strategies (e.g., antagonistic or synergistic interactions between components). Using the MOST framework allows us to optimize implementation support strategies for schools in three iterative phases – (1) screening, (2) refinement, and (3) testing. In the screening phase, we develop and select each discrete strategy to be optimized. In the refining phase, we decide the optimal dosage and combination of strategies by conducting the factorial experiment(s) and calculating the main effect of each strategy and interaction effects between strategies. In the testing phase, the optimal dosage and combination of strategies will be evaluated through a randomized controlled trial. This study protocol includes the first two phases of MOST in preparation for a future randomized controlled trial. The trial is reported following consort guidelines for a randomized trial, including extensions for clustered and factorial designs.

### 2.3 Study eligibility

For a school to be eligible to participate, it must be a public elementary school located in North or East Texas implementing the HSRC initiative (28, 29). For implementers to be eligible to participate, they must be 18 years old or older, be able to read and communicate in English, and be affiliated with a school that is eligible to participate.

For students to be eligible to participate in the study, they must be enrolled in the 5th or 6th grade, be at least 10 years old, have no known motor or cognitive impairments or other health conditions that would prevent them from completing assessments. The age of 10-years-old was selected because diagnostic criteria for MetS do not exist for children under that age (3). Students who have reported ever losing consciousness from pain or at the sight of blood will also be excluded to assist in diminishing the risk of fainting during assessments. Students must also be able to speak, read and write in English. For parents to be eligible to participate, they must be 18 years old or older with children enrolled in the participating schools and grade levels.

### 2.4 Procedure

#### 2.4.1 Texas A&M AgriLife Extension

Texas A&M AgriLife Extension is an education agency that provides programs, tools, and resources on a local and statewide level. AgriLife Extension has 250 offices (one in almost every Texas county), 900 extension educators, and a network of almost 100,000 volunteers that support the delivery of programs. Extension agents within the Family and Community Health and 4-H Youth Development units aim to help Texans better their lives through science-based educational programs designed to improve the overall health and wellness of individuals, families, and communities. Agents deliver programs in local communities (e.g., worksites, recreation centers, community events, military bases) as well as within local schools. Within schools, programs can either be delivered ad-hoc (i.e., schools choose one or more programs) or as part of the HSRC initiative.

#### 2.4.2 Healthy school recognized campus (HSRC) initiative

For this study, all schools will agree to participate in the HSRC initiative. The HSRC initiative promotes the delivery of physical activity and healthy eating programs by bundling AgriLife Extension’s school- and research-based programs. For a school to be recognized as a HSRC, they are required to host a school-wide walking challenge to increase students’ physical activity (i.e., Walk Across Texas), at least one other AgriLife Extension program for students, and at least one AgriLife Extension program for adults (examples in Table 1; full list of programs found at https://texas4h-hsrc.com/). Including both interventions for students and their caretakers (i.e., teachers or parents) is also a benefit of this bundled approach, as interventions that address more than one level of the ecological model (e.g., intrapersonal, interpersonal, organizational) can be more effective than single level interventions (30–32). Programs are delivered by one or more Extension agents (i.e., health educators) in the county where the school is located (33, 34), and once the initiative’s requirements are complete, schools receive recognition via a banner and/or at a local school meeting (e.g., school board, school health advisory council).

**Table 1:** Examples of healthy school recognized campus’ research-based programs.

Program	Description	Research
Youth		
Walk Across Texas! (Youth) (69, 70)	8-week program designed to help youth across Texas establish the habit of regular physical activity using a fun and motivating team approach.	- Doubled the number of steps taken over the course of the program. - Increased the amount of time that students were active with their parents. - Decreased BMI percentile.
Learn Grow Eat and Go! (70, 71)	10-week program that teaches students about gardening, healthy eating, and being active.	- Increased the number of vegetables tasted, vegetable preferences, and nutrition knowledge. - Decreased BMI percentile.
Choose Health: Food, Fun and Fitness (72–74)	6-session program that encourages healthy eating and fitness through hands-on activities and experiential education.	- Improved overall dietary intake, vegetable intake, fruit intake. - Reduced soda/fast food intake. - Read nutrition labels and share about healthy eating with families more often. - More likely to try new food. -Increased frequency of doing physical activities.
Color Me Healthy (75, 76)	9-week curriculum extension that focuses on healthy eating and physical activity via coloring, hands-on learning, and music to help children engage with their senses.	- Increased fruit and vegetable consumption - Increased students’ knowledge of healthy eating, knowledge about physical activity, and physical activity during the school day.
Play Streets (77–79)	Series of 1-day events with various activity stations that promote safe, fun, and active play.	- Encouraged physical activity participation - Provided a safe space for outdoor play - Fostered social interaction and community connection
Adult		
Walk Across Texas! (Adult) (80)	8-week program designed to help Texans establish the habit of regular physical activity using a fun and motivating team approach.	- Increased number of miles walked per week. - Decreased leisure time sitting.
Cooking Well with Diabetes (81)	3 to 4 lesson workshops that build skills toward planning, preparing, and cooking healthy meals.	- Increase fruit and vegetable intake and the use of healthy cooking methods (e.g., baking, broiling, grilling). - Decreased sugar-sweetened beverage intake.
Maintain No Gain (82)	6-week holiday challenge to help adults maintain their current weight between Thanksgiving and New Years. Weekly weigh-ins and exercise challenges are provided.	- A majority (~80%) of participants maintained or improved their weight status during the program.

#### 2.4.3 HSRC website

In addition to HSRC programming, all schools will receive access to the HSRC website^1^, which provides the research evidence for each program and shows the alignment of each program with Texas Essential Knowledge and Skills – curriculum standards for public schools in Texas. The website also provides step-by-step instructions on how to get recognized as a HSRC, a list of recognized schools, details on programs for different age groups, and resources. Resources include material such as physical activity guidelines, nutrition facts, mental health resources and positive youth development websites.

#### 2.4.4 School recruitment

We will work with Texas A&M AgriLife Extension agents to identify schools that may be interested in participating in the study. Specifically, schools that have previously participated in AgriLife’s physical activity or nutrition programs in the past, or that have recently expressed interest in receiving school-based health programs, will be contacted. We will also share information about HSRC with eligible schools via email, at school health conferences, or at events where school staff are present. Schools that agree to participate will sign a memorandum of understanding and a site authorization that permits the conduct of research on the school campus. Schools will also complete an application for the HSRC initiative. The application lists the initial AgriLife programs that schools can choose to complete during the next year; however, schools can change their programs at any point throughout the school year.

#### 2.4.5 Participant recruitment

We aim to recruit about 20 parents (320 total), 20 students (320 total), and 10 HSRC implementers (160 total) at each school (two cohorts of *n* = 8 schools) enrolled in the study. We will share recruiting materials online (e.g., email) and in-person (e.g., back-to-school events, student drop-off and pickup) to provide details about the study. Parents agreeing to participate will complete the consent form for their child and themselves, a short survey at pre- and post-intervention, and they will be compensated with a $10 gift card at each time point. The research team will meet in-person with children who have received signed parental consent to explain the study and answer any questions. Children will sign an assent form before participating in a survey and biometrics assessment at pre- and post-intervention. They will also receive an item worth $10–$15 (e.g., t-shirt, water bottle, active toy) for each data collection timepoint (pre- and post-intervention). Children who do not agree to participate in the study will still be allowed to participate in HSRC programs. HSRC implementers (e.g., Extension agents, school staff, administrators) will complete a consent form agreeing to participate, a survey pre- and post-intervention, and interviews following the intervention. They will be compensated with a $20 gift card for each survey and $50 for the interview.

#### 2.4.6 Randomization

In each of the two cohorts, schools will be randomized to one of eight experimental conditions and will receive a different combination of implementation strategies (Table 2). In each school year, four schools will receive each implementation strategy, and four schools will not (all schools will receive the website as a constant implementation strategy). Schools and data collectors will be blinded to randomized conditions until a school completes data collection. An interim analysis (following processes outlined below) will be conducted between the two cohorts to determine if implementation strategies should be removed and/or replaced, as well as if any of the current implementation strategies should be modified.

**Table 2:** Randomized experimental conditions for this study.

Experimental condition	Constant	Factor 1	Factor 2	Factor 3
1	Website/TEKS alignment	None	None	None
2	Website/TEKS alignment	None	None	Enhanced engagement opportunities
3	Website/TEKS alignment	None	School-to-school mentoring	None
4	Website/TEKS alignment	None	School-to-school mentoring	Enhanced engagement opportunities
5	Website/TEKS alignment	Additional resources	None	None
6	Website/TEKS alignment	Additional resources	None	Enhanced engagement opportunities
7	Website/TEKS alignment	Additional resources	School-to-school mentoring	None
8	Website/TEKS alignment	Additional resources	School-to-school mentoring	Enhanced engagement opportunities

Each of the 8 conditions will be randomized one time per cohort.

#### 2.4.7 Implementation support strategies

**Figure 1:**
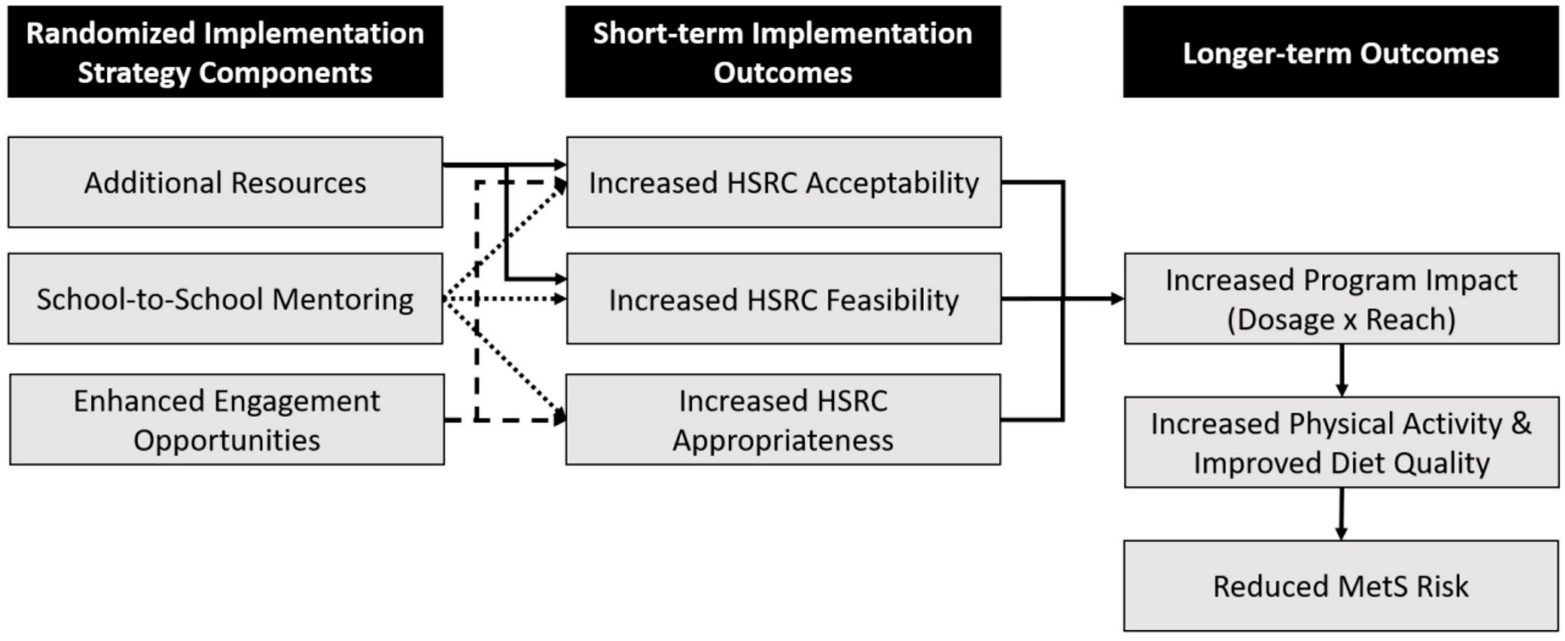
MOST logic model.

We selected and developed implementation strategies based on preliminary reports of barriers and facilitators to the use of the HSRC initiative (29, 35). The developed strategies are described below using Proctor’s recommendations to name, define, and specify implementation strategies when they are reported, as well as aligned with the StaRI reporting guidelines (36, 37). We also developed a Multiphase Optimization STrategy (MOST) logic model describing the randomized strategies’ hypothesized impact on implementation, behavioral, and health outcomes (Figure 1) (26, 38).

##### 2.4.7.1 Additional resources

We define the additional resources strategy as monetary support for participant incentives that could aid in improving program implementation outcomes (i.e., acceptability, feasibility). The research team will provide each Extension agent randomized to this strategy with a $2,000 budget to spend on incentives for student and adult participants in their HSRC programs – an amount based on our prior research. To streamline the process and provide example incentives for programs within HSRC – which was a recommendation from implementers – a password-protected page hosted on the HSRC website will feature an interactive form with some example incentive items and their estimated prices. Extension agents will work with their schools, access this page to view the items and complete an order request form for the items they would like. The research team then will reach out to confirm items, quantity, shipping address, total price, and estimated delivery date. Extension agents will be able to spend the $2,000 all at one time or spread out over the course of the year. They are also able to buy incentives beyond those included on the website, if they remain within their budget.

##### 2.4.7.2 School-to-school mentoring

We define the school-to-school mentoring strategy as the research team facilitating connections between schools that are implementing HSRC at the same time as one another to provide a support system and technical assistance to improve implementation outcomes (i.e., acceptability, appropriateness, feasibility) – a recommendation based on qualitative interviews and focus groups. The main components of this strategy will be: (1) an introductory email, (2) monthly meetings, (3) a private Facebook group, and (4) quarterly newsletters. Immediately following randomization, participating schools will receive an introductory email welcoming them to the school-to-school mentoring program and informing them about its components (e.g., the Facebook group, newsletters). Starting around November (1–2 months into the implementation process), the research team will host monthly virtual meetings with implementers to discuss the programs being completed and successes and barriers to program implementation. At the first meeting, implementers will be invited to join a private Facebook group, which will serve as a hub for HSRC program implementers to discuss ideas, share insights on their implementation process, and seek advice outside of the group meetings. Within the Facebook group, the research team will post prompts periodically to foster engagement with the page. Finally, schools will receive quarterly newsletters designed by the research team (up to four times during the year), which will include the following elements: (1) a previously recognized HSRC school, (2) links to helpful resources for program implementation, (3) a spotlighted HSRC program, (4) a calendar of local county health-focused events, and (5) information on AgriLife Extension and its agents.

##### 2.4.7.3 Enhanced engagement opportunities

We define enhanced engagement opportunities as physical activity and nutrition competitions encouraging schools and students to be active participants in HSRC programs to improve implementation outcomes (i.e., acceptability and appropriateness) – a strategy based on qualitative interviews and focus groups. More specifically, the two competitions were the “Walk Across Texas Mileage & Participation Contest” – aligned with the required Walk Across Texas program – and the “MyPlate Recipe Contest” – aligned with various nutrition programs within the HSRC initiative (e.g., Learn, Grow, Eat, and Go). For the Walk Across Texas Mileage & Participation Contest, students and school staff work toward two goals: (1) attain 10% adult participation in their Walk Across Texas event and (2) collectively accumulate an average of 26.2 walking miles per person – goals selected based on previous participation levels in HSRC programs. For each goal that schools meet, they earn a prize picked by the school’s principal (e.g., extra recess, no uniforms for a day). For the MyPlate Recipe Contest, students compete against one another and against other schools by crafting healthy, MyPlate-aligned recipes (e.g., use half-fruits and vegetables). Submissions will be evaluated for their nutritional value and creativity, and student winners will be awarded monetary prizes at each grade level. Additionally, the school with the highest percentage of student participation receives $500.

### 2.5 Outcomes

Baseline data collection (T1) will occur prior to randomization within the first 3 months of the academic year (Aug. 1 – Oct. 31). Post-test data collection (T2) will occur in the last 2 months of the academic year (April 1 – May 31). Data collection for parents and implementers consists of a survey, whereas data collection for students includes a survey and biometrics assessment. Parent and implementer surveys will be administered online via REDCap (an online survey platform), whereas student surveys and biometrics (i.e., objective measures of cardiovascular disease risk) will be collected during an in-person data collection event. A summary of constructs measured is included in Table 3.

**Table 3:** Summary of constructs measured.

Construct	Implementers	Students	Parents
Implementation outcomes			
Reach	P		
Dose delivered	P		
Acceptability, appropriateness, feasibility	O		
School characteristics	O		
Perceptions of healthy school recognized campus	O		
Effectiveness outcomes			
Metabolic syndrome		P	
Cardiovascular fitness		P	
Dermal carotenoids		P	
Body mass index		P	
Psychosocial determinants of physical activity and healthy eating		O	
Physical activity	O	O	O
Frequency of foods consumed	O	O	O
Engagement in the school and community		O	O
Sociodemographic characteristics	O	O	O

P, primary; O, other.

#### 2.5.1 Primary implementation outcomes

On the implementer survey, we will ask implementers which programs they chose to deliver, the classrooms that received those programs, the number of students in those classrooms, the number of sessions they delivered from those programs, and the normal session length for that program. In the student survey, we will ask the students to identify their teachers. From this data, each will be determined as the total number of students who received programs. We will also calculate the dose that each participant received (primary outcome) by multiplying the number of sessions by the session length and summing them across all programs that the students’ teachers received.

#### 2.5.2 Other implementation outcomes

To measure other variables in our logic model, the implementer survey will ask about the acceptability, appropriateness, and feasibility of implementing the HSRC initiative (39). We will also assess school characteristics related to the implementation of the HSRC initiative (e.g., school culture, leadership engagement, resources) using an existing measure of the inner setting from the Consolidated Framework for Implementation Research (40). Existing validated measures will also be modified to evaluate implementers’ perceptions of the HSRC initiative (e.g., knowledge, attitudes, and self-efficacy) (41). To determine if HSRC programming had any effect on implementer health behaviors, implementers (e.g., principals, teachers, extension agents, and support staff involved with HSRC’s delivery) will be asked about their physical activity and eating behaviors (42, 43).

#### 2.5.3 Primary effectiveness outcomes

Primary effectiveness outcomes include metabolic syndrome, cardiovascular fitness, fruit and vegetable consumption, and body mass index.

##### 2.5.3.1 Metabolic syndrome

The International Diabetes Federation defines MetS in children aged 10–16 years old as having a waist circumference above the 90th percentile for a child’s sex and age, and at least two of the four following criteria: triglycerides ≥ 1.7 mmol/L, HDL-C < 1.03 mmol/L, systolic blood pressure ≥130 mmHg or diastolic blood pressure ≥ 90 mmHg, and glucose ≥5.6 mmol/L or known diabetes (3). To assess MetS risk factors, we will measure waist circumference at the midpoint between the floating rib and iliac crest (44). For blood pressure, we will use an automated Omron sphygmomanometer and an appropriately sized cuff to measure blood pressure on children’s upper right arm after they have been seated for 5 min (45). Both waist circumference and blood pressure measurements will be taken up to three times, and the two closest measurements will be averaged. To measure triglycerides, HDL-C, and glucose, we will use a portable CardioChek Plus analyzer to collect a single capillary blood sample following an overnight fast. MetS will be evaluated as binary (i.e., yes or no) and continuous (i.e., number of MetS risk factors) outcomes.

##### 2.5.3.2 Cardiovascular fitness

We will determine cardiovascular fitness using the Progressive Aerobic Capacity Endurance Run (46). A research team member will lead the children through the test to provide instructions and pacing, after which, children will complete the test in groups of five or six. Children will run back and forth 20 m, with an initial running speed of 8.5 km/h and a progressive 0.5-km/h increase in running speed every minute thereafter. Number of laps completed will be used in conjunction with age, height, weight, and biological sex to estimate cardiovascular fitness (i.e., VO2 max) using a standardized estimation equation.

##### 2.5.3.3 Fruit and vegetable consumption

We will estimate children’s fruit and vegetable intake using a Veggie Meter, a non-invasive portable device that measures skin carotenoid levels (i.e., a biomarker of fruit and vegetable intake) (47). We will collect up to three readings per child and average the two closest measurements.

##### 2.5.3.4 Body mass index (BMI)

We will measure BMI by collecting children’s height using a Seca stadiometer and weight using a Tanita body composition analyzer. We will take up to three measurements and average the two closest measurements. We will calculate BMI percentile using the standard formula (kilogram per meter squared) and normative values from the Centers for Disease Control growth charts (48).

#### 2.5.4 Other student outcomes

On the student survey, we will assess several psychosocial constructs (e.g., attitudes, subjective norms, perceived behavioral control) related to physical activity and healthy eating (49–51). Using the Youth Activity Profile, we will also evaluate time spent being active at school, outside of school, and time spent being sedentary outside of school (52). Students will complete a food frequency questionnaire that asks about how frequently they consume 20 different foods and 10 drink categories, which will be used to calculate the SPAN survey’s Healthy Eating Index and its subscales (healthy and unhealthy food index) (53, 54). Finally, we will evaluate students’ engagement in the school and community and several other health-related topics (e.g., sleep) (55, 56).

#### 2.5.5 Parent outcomes

To determine if HSRC programming had any effect on parents’ health behaviors, they will complete the 16-item Mediterranean Eating Pattern for Americans screener asking about the frequency at which they eat and drink foods and beverages (57). They will also complete the International Physical Activity Questionnaire to evaluate physical activity in five domains: (1) occupational, (2) transportation, (3) housework, house maintenance, caring for family, (4) recreation, sport, leisure time, and (5) sedentary habits (58). Finally, we will ask several questions to evaluate their engagement in the school and community (59).

#### 2.5.6 Sociodemographic measures

Students’ sociodemographic measures will include age, date of birth, biological sex, grade level, race, and ethnicity. Staff’s sociodemographic measures will include their role implementing HSRC, the number of years they have worked at their school, and the number of years they have worked in education. Parents’ sociodemographic measures will include marital status, education, income, and employment.

### 2.6 Analysis

#### 2.6.1 Statistical and power analysis

This is an optimization study to screen and refine components of a support strategy for the HSRC initiative in preparation for a fully powered randomized controlled trial; thus, this study is not fully powered to examine differences in implementation or effectiveness outcomes (60). To analyze outcomes, we will use univariate and bivariate statistics to determine the distribution of outcome measures and to identify relevant covariates (61). To test between-implementation strategy differences on implementation and effectiveness outcomes, students at schools with and without each implementation strategy (independent variable) will be evaluated using a generalized linear model framework. Baseline scores will be added as a covariate (when applicable), post-intervention score is the outcome, and school is a clustering variable (62). Other covariates (e.g., school size), identified in the preliminary bivariate analyses, will also be included in the model. If missing data occurs, we will mitigate potential biases by analyzing multiple imputed datasets under an intention-to-treat approach (63). Analyses will be compared to per-protocol analyses (64). Interaction effects between strategies are normally small in behavioral trials; however, we will review plots of combinations of strategies to determine if a specific combination of strategies warrants further evaluation. If so, we will add an interaction term to our model. We will calculate standardized effect estimates for each strategy (i.e., Cohen’s *d*), compare effect estimates to make decisions about which components to include in the final support strategy, and report these estimates in our results.

As the goal of this project is to refine and optimize implementation strategies for testing in a future trial, we will conduct an interim analysis following the first cohort of schools using the approach outlined above. This analysis will be repeated following the second cohort of schools. Depending on the results of the interim analysis and potential changes to the strategies that result from the interim analysis, we will conduct a pooled analysis across both cohorts, unless the implementation strategies undergo substantive modifications between cohorts, in which case we will only report the individual results from each cohort.

## 3 Discussion

This study uses rigorous implementation science frameworks and methodologies to develop an implementation strategy for the HSRC initiative. In particular, the use of the MOST framework to develop, and a factorial design to test, the implementation strategies allow for the individual evaluation of three different discrete implementation strategies – additional resources, school-to-school mentoring, and enhanced engagement – and the understanding of potential interactions between these discrete strategies (26, 38). This is a substantial advancement within the field of implementation science, as most implementation strategy developers currently develop and test implementation strategies as packages (e.g., multiple strategies compared to control condition) (26, 38). Testing of implementation strategies as a package prevents understanding of how individual components within a package affect one another (e.g., antagonistic or synergistic interactions between components), which means that if the strategy needs to be adapted in the future (e.g., components removed), then another trial may be required, ultimately slowing the rate at which programs can be successfully delivered in practice. This study will provide effect estimates for each individual strategy and all combinations of strategies; thus, future researchers and practitioners will be able to use our outcomes to inform implementation of school-based interventions with seven different combinations of implementation strategies (i.e., three discrete strategies, three 2-way interactions, and one 3-way interaction), as opposed to the one effect estimate offered by a randomized controlled trial.

Implementation strategies that are used in this study were developed using school and community stakeholder input (e.g., extension agent) (29, 35). By using data from interviews and focus groups with local stakeholders and spending data from previous implementation efforts of HSRC (29, 35), we hope that we have developed strategies that are well-aligned with community needs and resources. Previous literature asserts that implementation strategies that are developed using stakeholder input are more impactful and equitable than implementation strategies that are developed without that type of input (65, 66). Given that the HSRC initiative is being implemented in a high cardiovascular disease risk area, we hope that obtaining and applying community stakeholder input in the development of the implementation strategies used in this study can improve the relevancy of these strategies to the local context. If so, this process may be replicated to improve the implementation of evidence-based interventions in other contexts.

We also evaluate implementation outcomes and effectiveness outcomes. By presenting a hypothesized logic model of the pathway between implementation strategies, implementation outcomes, and effectiveness outcomes, we can begin to understand how changes in short-term and long-term implementation outcomes affect program effect sizes for behavioral and health outcomes. Although current knowledge suggests that improved implementation of programs leads to improved health outcomes for participants, it is less clear which barriers to implementation are most important to address to improve implementation outcomes, which implementation outcomes have the biggest impact on improvements in effectiveness, and the strategies that can be used to address barriers. As previous literature demonstrates, there are at least 22 different barriers to the implementation of school-based programs (20, 21), over a dozen different implementation outcomes (67) and 75 different implementation strategies that can be used in the school setting (68). By proposing and evaluating how implementation strategies are related to barriers, implementation outcomes, and effectiveness outcomes, our work can begin to identify important pathways for improving the delivery and effectiveness of physical activity and healthy eating programs in the school setting.

Finally, the HSRC initiative bundles programs within the school setting to try to improve physical activity, healthy eating, and MetS risk among students. More specifically, all students at the school participate in an 8-week school-wide walking program (i.e., Walk Across Texas) (69, 70), and smaller groups of students at the school (e.g., physical education class, after-school club) participate in additional programming. This type of bundled approach is ideal for Extension agents, as once they start delivering programs at the school, it is often easier to implement additional programs within the school setting. Additionally, schools are an important setting to improve physical activity and diet quality among youth, as students spend an average of 6.5 h there each day for over 180 days each year (9), and school-based programs are an evidence-based approach for improving obesity outcomes (5–8). Still, given that bundled approaches have had mixed effectiveness in the clinical setting (13), it is unclear if this type of bundled approach has positive effects on students’ behavioral (i.e., physical activity and healthy eating) and health outcomes within the school setting. Thus, our study will evaluate changes in health outcomes over the course of one school year. Furthermore, as the HSRC initiative also requires that parents and/or school staff participate in a physical activity and healthy eating program – recommendations aligned with school health and ecological models (15–18)– we will evaluate physical activity and nutrition outcomes in these groups to determine if HSRC has any impact beyond students. These results can help to inform further modification to the way the HSRC initiative is designed and implemented within the school setting.

### 3.1 Conclusion

Overall, this study aims to evaluate the individual and combined (synergistic or antagonistic) impact of three implementation strategies on the delivery of bundled physical activity and nutrition programs and evaluate the effectiveness of this approach on improving MetS among elementary school aged children. By using rigorous implementation science frameworks, developing three implementation strategies – additional resources, school-to-school mentoring, and enhanced engagement – and evaluating implementation and effectiveness outcomes, this study aims to determine which implementation strategies have the most impact on improving the delivery of a school-based initiative to deliver multiple research-based physical activity and nutrition programs. The results from this study can inform researchers and practitioners on the appropriate processes for developing and evaluating the delivery of bundled programs within a school setting. Furthermore, the implementation strategy or combination of strategies that have the biggest impact on outcomes can be scaled up to improve the delivery of the HSRC initiative within other schools across Texas and the United States.

## Footnotes

^1^ https://texas4h-hsrc.com/
